# Another Dental College

**Published:** 1896-10

**Authors:** 


### Another Dental College.

During the past year a dental college has been organized in Pittsburgh, Pa., and apparently under good auspices. It is taking high ground in its requirements for entrance, in the curriculum, and in regard to graduation. Dr. J. G. Templeton is Dean, and he has a good corps of co-workers. The school will be heard from.

